# Effort-reward imbalance and common mental disorders among firefighters

**DOI:** 10.47626/1679-4435-2026-1638

**Published:** 2026-07-26

**Authors:** Alexandre Sérgio de Oliveira Angelin, Sérgio Roberto de Lucca

**Affiliations:** 1 Universidade Estadual de Campinas, Faculdade de Ciências Médicas, Campinas, São Paulo, Brazil

**Keywords:** firefighters, occupational stress, occupational health, mental health

## Abstract

**Introduction:**

Occupational stress and psychosocial factors at work are recognized as triggers of psychological distress, musculoskeletal disorders, and decreased functional capacity in the contemporary workplace.

**Objectives:**

This study investigated effort-reward imbalance and common mental disorders among 372 firefighters working in Brazil.

**Methods:**

This was a cross-sectional study conducted with a sample of 372 firefighters using a log-binomial regression model.

**Results:**

The prevalence of common mental disorders was 9.7% in the total sample and reached 10.7% in the high-risk group (effort-reward imbalance ratio > 1). Log-binomial regression showed that the effort-reward imbalance model increased the explanatory power of mental distress to 12.1%. High effort was associated with an increased risk of common mental disorders (prevalence ratio = 2.21), whereas overcommitment had the greatest impact on prevalence (prevalence ratio = 2.41). Effort was also the main predictor of symptom severity (β = 0.443; p < 0.001).

**Conclusions:**

These findings indicate that effort-reward imbalance constitutes an important marker of psychosocial risk among firefighters. The population attributable fraction for overcommitment was 51.1%, indicating that more than half of common mental disorder cases may be attributed to this dimension. Thus, occupational demands and perceived recognition emerged as key determinants of mental health in this professional category. The study reinforces the need for mental health surveillance and promotion strategies that consider the specific characteristics of firefighters’ military operational environment.

## INTRODUCTION

Occupational stress and psychosocial factors at work are recognized as triggers of psychological distress [1], musculoskeletal disorders [2], and reduced functional capacity in the contemporary workplace. They also constitute a major public health concern on a global scale, particularly among public safety professionals [3]. It is estimated that 15% of the economically active population worldwide experience some form of mental disorder resulting from occupational stress at some point during their working lives. This finding highlights the workplace as a central setting for the production of illness [4].

The professional category of firefighters is characterized by high work demands, recurrent exposure to high-risk situations, and decision-making under extreme conditions [5]. Although these professionals exhibit a low prevalence of formal mental disorder diagnoses, they frequently present signs of fatigue, emotional exhaustion, and psychosomatic symptoms [6]. Institutional stigma and self-stigma, by hindering help-seeking behaviors, contribute to the underreporting of mental distress among firefighters [7].

When unrecognized, illness increases the risk of accidents, work absences, early retirement, and/or failures in emergency response, thereby constituting a strategic problem for public safety and civil defense systems [8]. Most investigations on mental health focus on standardized clinical diagnoses, neglecting subclinical manifestations that are epidemiologically relevant [9]. Although relevant, these manifestations do not meet formal psychiatric diagnostic criteria. They include symptoms such as insomnia, fatigue, irritability, and somatic complaints. These conditions are traditionally grouped under the category of common mental disorders (CMD) [10].

Screening instruments such as the Self-Reporting Questionnaire-20 (SRQ-20) [11] are widely used in epidemiological studies to capture the spectrum of mental distress. These instruments are particularly useful in contexts where stigma and organizational culture hinder the expression of psychological suffering [12], as they identify broad and nonspecific manifestations. The effort-reward imbalance (ERI) model, proposed by Siegrist [13], assesses the extrinsic dimensions of work (effort, reward, and overcommitment) and allows the analysis of complex psychosocial mechanisms associated with psychological illness [14].

Applied to the reality of firefighters, this model makes it possible to investigate two central questions: i) to what extent does the perceived imbalance between effort and reward at work act as a determining psychosocial factor for the presence of symptoms of mental illness? And ii) does overcommitment act as an amplifying factor in this association, increasing vulnerability to psychological distress?

Accordingly, the current study aims to analyze the prevalence of ERI and CMD, as well as to investigate how high effort and low reward contribute to mental distress among firefighters.

## METHODS

A cross-sectional epidemiological study was conducted with firefighters from the units of Regional Fire Command-1 (CBI-1), the agency responsible for the Campinas region, São Paulo State, Brazil. The total workforce in the region comprises approximately 1,300 active firefighters, distributed between the administrative sector (~450 firefighters) and the operational sector (~950 firefighters).

All active firefighters affiliated with CBI-1 were considered eligible for inclusion. Those on medical leave, vacation, or absent for other reasons related to their own health or that of family members were excluded (n = 58).

All eligible participants were invited to participate in the study, of whom 401 (30.84%) agreed to participate by signing the informed consent form. Participants who did not fully complete the electronic questionnaire were excluded (n = 29), resulting in a final sample of 372 participants (28.61% of the total eligible population). Data were collected between September 2025 and January 2026.

Access to participants was granted upon authorization from the CBI-1 command. Data were collected through the Google Forms^®^ platform, whose link was distributed by the Human Resources department of CBI-1 to all active firefighters in the institution. Researchers received the responses in anonymized form. To reduce sample losses, three additional contacts were made with the Human Resources department to encourage firefighter participation.

The research questionnaire consisted of three sections: i) participants’ sociodemographic and professional information (age group, sex, race/skin color, marital status, educational level, rank, operational role, and workplace); ii) the ERI model, short version validated for Brazilian Portuguese [15]; and iii) the SRQ-20 [12].

The ERI consists of 23 items rated on a four-point Likert scale (1 = disagree; 4 = strongly agree) and assesses the dimensions of effort (six items), reward (11 items), and overcommitment (six items). To analyze imbalance, the effort/reward ratio was calculated by dividing the effort score by the reward score and multiplying the result by a correction factor of 0.5454. This factor was used to compensate for the difference in the number of items between the scales [13].

The SRQ-20 was used to assess suspected CMD. This instrument was developed by the World Health Organization to screen for neurotic disorders and psychological distress in epidemiological studies [11]. Validated for the Brazilian population, SRQ-20 screens for physical and psychological symptoms experienced over the previous 30 days [12]. In this study, a cutoff point of ≥ 7 was adopted for both sexes, a criterion indicating suspected non-psychotic mental disorder and psychological distress, as recommended by Santos et al. [16] in studies involving police officers.

Statistical analysis was structured into three complementary stages. In the first stage, the sociodemographic, occupational, and psychometric profile of the sample was characterized. A significance level of 5% (α = 0.05) and 95%CIs were adopted.

The second stage consisted of bivariate analyses to explore associations between ERI dimensions and CMD assessed by the SRQ-20. Fisher’s exact test was used to evaluate associations between categorical variables, such as sex and suicidal ideation (item 17 of the SRQ-20).

Finally, the third stage comprised multivariable log-binomial regression analyses, with models adjusted for age, sex, and rank, aiming to isolate the effect of work organization and identify predictors of the studied outcomes. The independent variables included in the models were sex and ERI dimensions, whereas CMD constituted the primary outcome.

Selection of the final model was based on the corrected Akaike information criterion (AICc) and the absence of multicollinearity (variance inflation factor < 5). Population attributable fractions (PAF) were calculated from the regression models to quantify the impact of occupational factors on mental illness. Additionally, multiple correspondence analysis (MCA) was performed to visually synthesize the relationships between professional profiles and CMD. All data were processed using IBM SPSS Statistics for Windows, version 22.0 (IBM Corp., Armonk, N.Y., USA).

The study was approved by the Human Research Ethics Committee of the School of Medical Sciences, Universidade Estadual de Campinas (CAAE No. 86128224.4.0000.5404).

## RESULTS

The sample comprised 372 military firefighters (Table 1). Most participants were male (87.68%; n = 326), aged 35-44 years (38.44%; n = 126), married or living in a stable union (69.62%; n = 259), and self-identified as White (73.40%; n = 273). Approximately one-third of the participants had completed higher education (32.53%; n = 121). Enlisted personnel (privates, corporals, sergeants, and warrant officers) represented the largest proportion of the sample (94.62%; n = 352). Operational activities were predominant (66.40%; n = 247), and approximately half of the participants worked in Campinas (51.34%; n = 191).

**Table 1.** Sociodemographic profile and psychosocial work indicators (ERI model) associated with the prevalence of CMD (n = 372). Campinas, Brazil, 2026

Variables	ERI				Effort				Reward				Overcommitment				Prevalence of CMD	
	At risk		Not at risk		High		Low		High		Low		High		Low			
Total (n = 372)	(n)	(%)	(n)	(%)	(n)	(%)	(n)	(%)	(n)	(%)	(n)	(%)	(n)	(%)	(n)	(%)	(n)	(%)
**Age group, years**	**164**	**44.09**	**208**	**55.91**	**183**	**49.20**	**189**	**50.80**	**199**	**53.50**	**173**	**46.50**	**166**	**44.60**	**206**	**55.40**	**36**	**9.70**
18-24		16.70		83.30		20.00		80.00		45.00		55.00		10.00		90.00		0.00
25-34		44.60		55.40		56.80		43.20		45.30		54.70		48.40		51.60		6.30
35-44		42.00		58.00		53.10		46.90		58.70		41.30		49.70		50.30		15.40
45-54		44.30		55.70		42.60		57.40		56.40		43.60		43.60		56.40		7.90
55-64		33.30		66.70		46.20		53.80		46.20		53.80		23.10		76.90		0.00
> 65		0.00		0.00		0.00		0.00		53.50		46.50		0.00		55.40		0.00
**Sex**																		
Female		51.20		48.80		50.00		50.00		34.10		65.90		36.40		63.60		22.40
Male		40.70		59.30		49.40		50.60		55.80		44.20		45.40		54.60		8.00
Not reported		0.00		100		0.00		100		100		00.00		100		0.00		0.00
**Self-reported race/skin color**																		
White		39.30		60.70		46.90		53.01		54.90		45.10		41.40		58.60		9.50
Asian		66.70		33.30		66.70		33.30		50.00		50.00		83.30		16.70		16.70
Brown		46.50		53.50		54.10		45.90		52.70		47.30		48.60		51.40		9.50
Indigenous		0.00		100		0.00		100		100		0.00		100		0.00		0.00
Black		52.90		47.10		61.10		38.90		33.30		66.70		61.10		38.90		11.10
Prefer not to disclose		0.00		0.00		0.00		0.00		0.00		0.00		0.00		0.00		0.00
**Marital status**																		
Single		37.00		63.00		44.70		55.30		50.60		49.40		42.40		57.60		9.40
Married/Stable union		41.10		58.90		49.40		50.60		53.70		46.30		44.40		55.60		10.40
Divorced/Separated		60.70		39.30		60.70		39.30		60.70		39.30		53.60		46.40		3.60
Widowed		0.00		0.00		0.00		0.00		0.00		0.00		0.00		0.00		0.00
**Educational attainment**																		
Completed secondary education		38.30		61.70		41.10		58.90		46.70		53.30		36.70		63.30		11.10
Completed technical education		36.80		63.20		47.40		52.60		50.00		50.00		52.60		47.40		10.50
Completed higher education		39.70		60.30		48.80		51.20		60.30		39.70		43.80		56.20		11.60
Incomplete higher education		46.70		53.30		52.20		47.80		47.80		52.20		41.30		58.70		0.00
Lato sensu graduate degree (specialization)		49.30		50.70		58.90		41.10		56.20		43.80		54.80		45.20		11.00
Stricto sensu graduate degree (Master’s)		25.00		75.00		50.00		50.00		50.00		50.00		25.00		75.00		0.00
Stricto sensu graduate degree (Doctorate)		0.00		0.00		0.00		0.00		0.00		0.00		0.00		0.00		0.00
**Rank**																		
Private		37.80		62.20		46.70		53.30		53.30		46.70		39.00		61.00		8.60
Corporal		47.10		52.90		44.40		55.60		47.20		52.80		47.20		52.80		13.40
Sergeant		41.40		58.60		55.40		44.60		63.00		37.00		43.50		56.50		4.30
Warrant Officer		58.30		41.70		69.20		30.80		61.50		38.50		46.20		53.80		7.70
Officer Cadet/Officer Trainee		0.00		0.00		0.00		0.00		0.00		0.00		0.00		0.00		25.00
Lieutenant		0.00		100		75.00		25.00		100		0.00		75.00		25.00		10.00
Captain		30.00		70.00		50.00		50.00		20.00		80.00		60.00		40.00		0.00
Major		0.00		0.00		0.00		0.00		0.00		0.00		0.00		0.00		0.00
Colonel/Lieutenant Colonel		0.00		100		0.00		0.00		0.00		100		0.00		100		0.00
**Duty status**																		
Administrative		36.20		63.80		44.30		55.70		55.70		44.30		41.00		59.00		7.40
Operational		44.80		55.20		52.20		47.80		52.20		47.80		46.20		53.80		10.10
Removed from operational duties		0.00		100		0.00		100		66.70		33.30		66.70		33.30		66.70
**Operational unit**																		
7th FB		32.60		67.40		51.70		48.30		61.80		38.20		40.40		59.60		6.70
15th FB		43.80		56.30		52.90		47.10		62.70		37.30		43.10		56.90		25.50
16th FB		50.50		49.50		43.60		56.40		46.50		53.50		46.50		53.50		0.00
19th FB		36.80		63.20		35.00		65.00		40.00		60.00		50.00		50.00		15.00
CBI-1		43.80		56.30		40.00		60.00		55.00		45.00		35.00		65.00		0.00
CCB		40.40		59.60		56.00		44.00		50.50		49.50		48.40		51.60		15.40

CBI-1 = regional fire command-1; CCB = fire department command; CMD = common mental disorders; ERI = effort-reward imbalance; FB = fire brigade.

Women accounted for 11.80% of the sample (n = 44), with the predominant characteristics being age between 35 and 44 years (n = 17), married or living in a stable union (n = 21), completed secondary education (n = 14), self-identification as White (n = 34), the rank of private (n = 14), and employment in administrative activities (n = 23).

With regards to ERI, high risk (ERI ratio > 1) was identified in 44.09% of firefighters. Concerning effort, 49.20% reported high effort and 50.80% low effort. Most participants (53.50%) reported high levels of reward. High overcommitment was observed in 44.60% of the sample, whereas 55.40% presented low overcommitment.

The 25-34-year age group showed the highest percentages of high effort (56.80%; n = 54), ERI risk (44.60%; n = 56), and overcommitment (48.40%; n = 46). Single military personnel exhibited higher frequencies of low overcommitment (57.60%; n = 49), whereas those who were married or living in a stable union reported greater perceptions of high reward (53.70%; n = 120).

Variations were observed across ranks and positions. Warrant officers showed the highest frequency of ERI risk (58.30%). Lieutenants exhibited the highest prevalence of high effort (75.00%), whereas captains showed the highest prevalence of low reward (80.00%).

When military units were analyzed, the 16th Fire Brigade (FB) showed the highest percentages of ERI risk (50.50%; n = 50) and high effort (43.60%; n = 44). The CBI-1 unit stood out for its high overcommitment (65.00%; n = 13).

When results were stratified by sex, most men were classified in the no ERI risk category (59.30%; n = 185), with a predominance of low effort (50.60%; n = 165). Women, in contrast, showed a higher frequency of ERI risk (51.20%; n = 22), low reward (65.90%; n = 29), and more critical indicators of psychosocial vulnerability, a finding similar to that reported among healthcare professionals [17] (Table 1).

Regarding the prevalence of suspected CMD, the overall sample presented a prevalence of 9.70% (n = 36), considering the SRQ-20 cutoff point of ≥ 7. Psychological distress was not homogeneously distributed across the sample. The highest prevalences were observed among participants aged 35-44 years (15.40%; n = 22) and those with incomplete higher education (11.60%; n = 8). Regarding occupation, the rank of corporal stood out among operational categories, with a CMD prevalence of 13.40% (n = 19).

The most critical indicator in the sample was observed among military personnel removed from operational activities but not from the fire station, whose CMD prevalence reached 66.70% (n = 2). Among military units, the highest prevalence of CMD was observed in the 15th FB (25.50%; n = 13), followed by personnel assigned to the Fire Department Command (15.40%; n = 14).

Sex-stratified analyses revealed significant disparities. Among men, the prevalence of CMD was 8.00% (n = 26), a value lower than the sample average. Among women, the prevalence was 22.40% (n = 10), indicating greater vulnerability to psychological distress (Table 1).

In the analysis of suicidal ideation prevalence over the previous 30 days (question 17 of the SRQ-20), 6.22% of participants (n = 23) responded affirmatively. When the results were stratified by sex, a marked disparity was observed between women (22.79%; n = 10) and men (4.01%; n = 13). Fisher’s exact test confirmed a statistically significant association, indicating that women exhibited a 7.09-fold higher prevalence of suicidal ideation compared with men (95%CI: 2.89-17.24).

### CORRELATIONS BETWEEN STRESSORS AND MENTAL HEALTH

**Figure 1.**
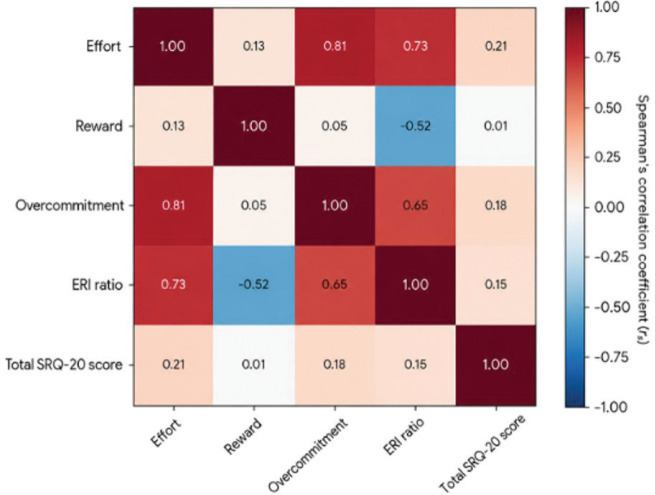
Correlation matrix (heatmap) between ERI dimensions and CMD assessed by the SRQ-20.

To explore the strength of the association between occupational stress dimensions and symptoms of psychological distress, Spearman’s correlation coefficient and a heatmap were used (Figure 1). Overall, a positive and statistically significant correlation was observed between the ERI ratio (the overall indicator of the model) and the total CMD symptom score (rs = 0.146; p < 0.005).

CMD = common mental disorders; ERI = effort-reward imbalance; SRQ-20 = Self-Reporting Questionnaire-20.

When the ERI dimensions were analyzed separately, effort showed the strongest correlations with mental health, being significantly associated with all SRQ-20 domains. A positive association was observed between effort and reward (rs = 0.215; p < 0.001) as well as between effort and the total SRQ-20 score (rs = 0.212; p < 0.001). Overcommitment was also positively associated with the total SRQ-20 score (rs = 0.180; p < 0.001).

### ASSOCIATION BETWEEN OCCUPATIONAL STRESS, SEX, AND MENTAL HEALTH

To identify factors associated with suspected CMD, log-binomial regression using incremental models was performed (Table 2), adopting suspected CMD (SRQ-20 ≥ 7) as the dichotomous dependent variable. Results were expressed as prevalence ratios (PRs).

**Table 2.** PR and 95%CI for CMD (SRQ-20 ≥ 7) according to log-binomial regression models (n = 372). Campinas, São Paulo, Brazil, 2026

Independent variables	Model 1 (ERI) PR (95%CI)	Model 2 (ERI + overcommitment) PR (95%CI)	Model 3 (adjusted*) PR (95%CI)
**Overcommitment**			
Low (Ref.)	1.00	1.00	1.00
High	—	2.65 (1.18-5.95)*	2.41 (1.09-5.34)*
**Effort**			
Low (Ref.)	1.00	1.00	1.00
High	2.19 (1.16-4.14)*	1.31 (0.63-2.73)	2.21 (0.93-5.23)
**Reward**			
High (Ref.)	1.00	1.00	1.00
Low	1.19 (0.60-2.35)	0.85 (0.41-1.75)	1.19 (0.65-2.17)
**Sex**			
Male (Ref.)	—	—	1.00
Female	—	—	3.77 (1.85-7.69)^†^
**Goodness of fit**			
AICc	169.86	165.83	159.76

AICc = corrected Akaike information criterion; CMD = common mental disorders; ERI = effort-reward imbalance; PR = prevalence ratio; Ref. = reference category; SRQ-20 = Self-Reporting Questionnaire-20.

The normality of continuous variables was assessed using the Kolmogorov-Smirnov test.

Model 3 adjusted for age group, sex, and unit/major command.

Log-binomial regression was used to estimate the PRs for the dichotomous outcome CMD (SRQ-20 ≥ 7). The adjusted model showed the best fit (AICc = 159.76).

* p < 0.05

† p < 0.001

As shown, effort emerged as the strongest positive predictor of symptom severity (β = 0.443; p < 0.001), indicating that the psychophysical burden inherent to firefighting constitutes an important determinant of psychological distress.

In the crude model (Model 1), ERI (ERI ratio > 1) showed a significant association with CMD, indicating that military personnel exposed to disrupted social reciprocity at work had a 2.19-fold higher prevalence of CMD (PR = 2.19; 95%CI: 1.16-4.14).

In the partially adjusted model (Model 2), the inclusion of overcommitment improved model fit (AICc = 165.83), revealing that excessive work involvement substantially increased the risk of mental illness (PR = 2.65; 95%CI: 1.18-5.95). In the final adjusted model, individuals with high overcommitment maintained a 2.41-fold higher prevalence of CMD (PR = 2.41; 95%CI: 1.09-5.34).

Simultaneously, sex emerged as the variable with the greatest magnitude of association in the study, with CMD prevalence being 3.77 times higher among women (95%CI: 1.85-7.69). The model adjusted for sociodemographic and occupational variables showed the best fit (AICc = 159.76), indicating that women exhibited a 3.77-fold higher prevalence of CMD compared with men in this sample (Table 2).

To translate individual risk into the institutional dimension, the PAF was calculated, as described in previous studies [18]. The PAF for overcommitment was 51.1%, indicating that more than half of CMD cases could be attributed to this dimension. In turn, ERI accounted for 34.4% of the mental distress observed in the sample.

**Figure 2.**
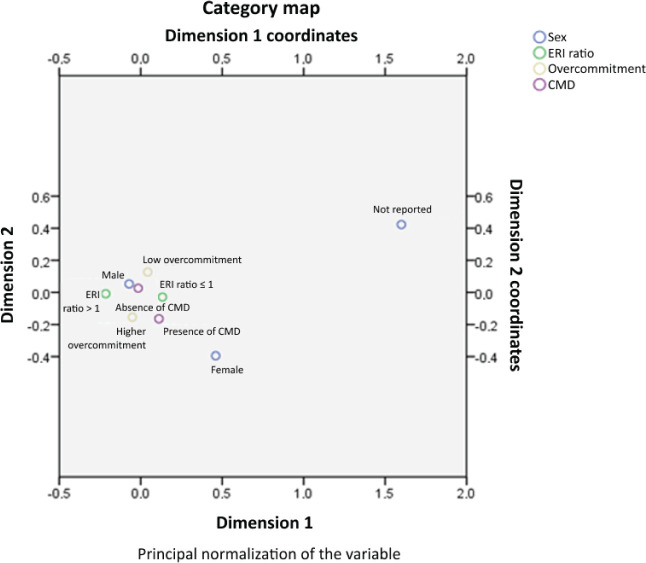
MCA map of sex, ERI, overcommitment, and CMD.

To visually illustrate these associations, MCA geometrically mapped the proximity between sociodemographic profiles, ERI, and health outcomes (Figure 2). The map revealed a clear polarization in the two-dimensional space, identifying two distinct profiles. On one side, a cluster was observed between female sex, high ERI (ERI ratio > 1), and suspected CMD. In the opposite quadrant, proximity was observed among male sex, occupational balance, and the absence of mental distress.

CMD = common mental disorders; ERI = effort-reward imbalance; MCA = multiple correspondence analysis.

## DISCUSSION

Integrating the findings, the ERI model emerged as an important theoretical framework for understanding mental distress among firefighters. Although effort, considered in isolation, was associated with CMD in the crude model (PR = 2.19; 95%CI: 1.16-4.14), this association lost statistical significance in the final model adjusted for overcommitment and sex (PR = 2.21; 95%CI: 0.93-5.23).

This finding suggests that the effect of effort on CMD may be partially mediated or overlapped by overcommitment (rs = 0.811; p < 0.001), together with sex. Thus, the internalization of occupational demands and sex-related vulnerability emerge as important factors associated with mental illness. Reward did not demonstrate an independent protective effect (rs = 0.007; p = 0.888), suggesting that, in military contexts, the absence of fair reciprocity may intensify the impact of effort, although its presence alone is insufficient to neutralize it. Therefore, low reward appears to amplify imbalance when present, but not to attenuate it when absent.

### PREVALENCE OF CMD AND THE HEALTHY WORKER EFFECT

The overall prevalence of suspected CMD (9.70%; n = 36) may have been underestimated due to the healthy worker effect and the culture of hyper-readiness typical of military organizations [19]. Professionals on medical leave were excluded from the sample (n = 58), as were individuals who may have declined participation because of stigma. Thus, the true magnitude of psychological distress in this occupational group may be greater than observed.

Despite this potential underestimation, the prevalence found was lower than that reported among primary healthcare workers in Salvador (21%) [17], but similar to that observed among military police officers in Rio de Janeiro [19]. This finding suggests that, although exposed to adverse working conditions, military firefighters may exhibit patterns of distress distinct from those of other occupational groups, influenced by institutional culture and stigma.

However, the results indicate that psychological illness is not homogeneously distributed within the organization, but rather intersects with social markers such as sex, rank, and race/skin color [17,20].

The PR of 3.77 for female sex reveals that women face a risk of illness nearly four times higher than that of men, even under the same operational demands. This estimate exceeds findings from national studies using the ERI model, such as those by Chor et al. [15] among university employees (rs ~0.50) and Sousa & Araújo [21] among healthcare workers. Therefore, it becomes imperative to treat gender as a marker of psychosocial vulnerability in organizational settings.

Consistent with Oliveira & Araújo [17], this vulnerability suggests that social reciprocity at work is weakened for women, as operational demands affect female personnel differently.

Female officers experience stress arising from decision-making responsibilities, whereas enlisted personnel face high operational demands and limited control over work processes. According to Dejours’ Psychodynamics of Work [22], the organization’s hierarchical structure establishes specific parameters for task negotiation, which may intensify suffering, as questioning working conditions is often perceived as hierarchical deviation rather than a warning sign for health.

As highlighted by Laurence & Matthews [23], human performance in military settings is conditioned by the interaction between individual capacities and occupational demands. The application of androcentric standards in activities requiring high physical performance may generate distinct burdens across genders. Furthermore, this burden is not distributed equally when race/skin color is considered [24]. The need to demonstrate exceptional performance to avoid stigmas of incompetence [23] is reflected in the present findings.

Black women are subjected to hypervigilance and hypervisibility [24], with institutional demands intensified by racial stereotypes. The Black racial group showed the highest ERI levels (52.90%) and high effort (61.10%). For these professionals, biopsychosocial burden is tripled, whether due to the physiological effort inherent to the occupation [25], gender barriers [17], or the pressure of representing a minority perceived as having no right to error or vulnerability [26].

According to Siegrist’s intrinsic activation hypothesis [13], overcommitment acts as an amplifier of strain, transforming external pressure into a chronic physiological response and increasing allostatic load [27]. This phenomenon reflects what Deleuze [28] defines as continuous control. Firefighters internalize the demand for readiness to such an extent that effort ceases to be perceived as external pressure and instead becomes understood as a personal imperative.

The PAF of 51.1% demonstrates that CMD cases arise from this internalized self-demand, considering that the military hierarchical structure may hinder the recognition of symptoms manifested as somatization or forced resilience [23]. From an occupational public health perspective, this PAF indicates that, even if all other working conditions remained unchanged, eliminating overcommitment among firefighters could reduce CMD cases by more than half (51.1%).

The disparity between sexes reached its most critical level in suicidal ideation, with a prevalence 7.09 times higher among women. This finding points to the work environment as a factor of extreme clinical severity, demanding qualitative investigations into women’s daily experiences within military settings. In a culture of hegemonic masculinity, somatization emerges as a socially acceptable language of suffering.

The results reveal that female firefighters share the same age range (35-44 years) and operational readiness regime as their male counterparts, while also facing additional stressors, such as work-family conflict and gender barriers [29,30]. Organizational culture values loyalty and sacrifice, which may create pressure to perform an identity grounded in invulnerability. In this context, resilience and action within militarism take on the character of overload for the female minority (12.32% of the workforce) (Table 1).

The discussion highlights the urgent need to transition toward Occupational Health surveillance strategies that recognize firefighters as a non-universal occupational category and acknowledge that the risk of illness is shaped by the position individuals occupy within the organization. Gender equity and the reform of processes that generate overload should be prioritized. Failure to recognize that female enlisted personnel are exposed to distinct psychological burdens may contribute to the perpetuation of health inequalities.

The sense of duty involves the internalization of extrinsic demands. Professional obligation transcends the technical sphere and occupies a moral dimension, in which individuals internalize total availability as a condition for belonging. Under this normalization of excess, firefighters may fail to prioritize signs of fatigue, operating according to a logic of invulnerability and suppression of suffering [19].

The paradox of operational readiness is supported by the impact of high effort (PR = 2.21; 95%CI: 0.93-5.23) in the adjusted model, demonstrating that the readiness required by the profession potentiates the risk of CMD. Although the ERI model establishes reward as a central axis, in the context of firefighting its manifestation may assume less visible and more internalized forms, an aspect deserving greater attention within military institutions.

### LIMITATIONS OF THE STUDY

The cross-sectional design of this study precludes the establishment of direct causal relationships. Furthermore, the use of a convenience sample from a specific region requires caution when generalizing the findings, particularly to firefighters from other regions of the country. In addition, the non-participation of individuals experiencing high levels of psychological distress may have led to an underestimation of the true prevalence of the outcome. To minimize these biases, future studies should include larger samples of firefighters, incorporate administrative records of leave due to CMD, and compare these data with mental health screening instruments.

## CONCLUSIONS

This study identified the prevalence of CMD and the impact of ERI on the mental health of Brazilian firefighters working in Campinas. The prevalence of CMD (9.7%), the paradox of effort — whose association lost significance in the adjusted model — and the disparity between sexes (PR = 3.77 and a higher prevalence of suicidal ideation among women) indicate that overcommitment plays a central role in the dynamics of psychological distress, with a PAF of 51.1%.

Recognizing that mental health is influenced by systemic factors reinforces the importance of adjustments in task design and work organization to preserve operational capacity and the long-term sustainability of the workforce. This study highlights the need for surveillance strategies aimed at task redesign, organizational justice, and gender equity.

## Data Availability

The data are available in a repository Mendeley Data (https://data.mendeley.com/drafts/gc74f8whjw).

## References

[1] Gomes PB (2022). evidências da relação trabalho e transtorno
mental no Brasil: uma revisão de escopo. Pensar Acad..

[2] Khoshakhlagh AH, Sulaie SA, Yazdanirad S, Orr RM, Laal F (2024). Relationships between job stress, post-traumatic stress and
musculoskeletal symptoms in firefighters and the role of job burnout and
depression mediators: a bayesian network model. BMC Public Health.

[3] Futino RS, Delduque MC (2020). Saúde mental no trabalho de segurança
pública: estudos, abordagens e tendências da
produção de conhecimento sobre o tema. Cad Ibero Am Direito Sanit..

[4] Souza HA, Bernardo MH (2019). Prevenção de adoecimento mental relacionado ao
trabalho: a práxis de profissionais do Sistema Único de
Saúde comprometidos com a saúde do trabalhador. Rev Bras Saude Ocup..

[5] Sokoloski ML, Rigby BR, Bachik CR, Gordon RA, Rowland IF, Zumbro EL (2020). Changes in health and physical fitness parameters after six
months of group exercise training in firefighters. Sports (Basel).

[6] Gulliver SB, Zimering RT, Knight J, Morissette SB, Kamholz BW, Pennington ML (2021). A prospective study of firefighters’ PTSD and depression
symptoms: the first 3 years of service. Psychol Trauma..

[7] Kim MJ, Jeong Y, Choi YS, Seo AR, Ha Y, Seo M (2019). The association of the exposure to work-related traumatic events
and work limitations among firefighters: a cross-sectional
study. Int J Environ Res Public Health.

[8] Coimbra MAR, Ferreira LA, Araújo APA (2020). Impactos do estresse na exposição ocupacional de
bombeiros: revisão integrativa. Rev Enferm UERJ..

[9] Seligmann-Silva E (2022). Trabalho e desgaste mental: o direito de ser dono de si mesmo.

[10] Witt M, Stelcer B, Czarnecka-Iwańczuk M (2018). Stress coping styles in firemen exposed to severe
stress. Psychiatr Pol..

[11] World Health Organization (1994). A user’s guide to the self-reporting questionnaire (SRQ)
[Internet].

[12] Mari JJ, Williams P (1986). A validity study of a psychiatric screening questionnaire
(SRQ-20) in primary care in the city of Sao Paulo. Br J Psychiatry.

[13] Siegrist J (1996). Adverse health effects of high-effort/low-reward
conditions. J Occup Health Psychol..

[14] Coelho D, Yamaguchi S, Harb A, Souza-Talarico JN (2024). Effort-reward and overcommitment at work and psychiatric symptoms
in healthcare professionals: the mediation role of allostatic
load. Compr Psychoneuroendocrinol..

[15] Chor D, Werneck GL, Faerstein E, Alves MGDM, Rotenberg L (2008). The Brazilian version of the effort-reward imbalance
questionnaire to assess job stress. Cad Saude Publica..

[16] Santos KO, Carvalho FM, de Araújo TM (2016). Internal consistency of the self-reporting questionnaire-20 in
occupational groups. Rev Saude Publica..

[17] Oliveira AMN, Araújo TM (2018). Situações de desequilíbrio entre
esforço-recompensa e transtornos mentais comuns em trabalhadores da
atenção básica de saúde. Trab Educ Saude..

[18] Rajnoveanu AG, Rajnoveanu RM, Motoc NS, Postolache P, Gusetu G, Man MA (2022). COPD in firefighters: a specific event-related condition rather
than a common occupational respiratory disorder. Medicina (Kaunas).

[19] Minayo MCS, Souza ER, Constantino P (2012). Missão prevenir e proteger: condições de vida,
trabalho e saúde dos policiais militares do Rio de Janeiro.

[20] Shi J, Chen Y, Li X, An Y (2021). Predicting posttraumatic stress and depression symptoms among
frontline firefighters in China. J Nerv Ment Dis..

[21] Sousa CC, Araújo TM (2024). Combined effects of gender, race, and occupational stressors on
mental health. Rev Bras Saude Ocup..

[22] Dejours C (2008). A loucura do trabalho: estudo de psicopatologia do trabalho.

[23] Laurence JH, Matthews MD (2012). The Oxford handbook of military psychology.

[24] Rando S (2022). Pensamento feminista negro: conhecimento, consciência e a
política do empoderamento.

[25] Pacholok S (2013). Into the fire: disaster and the remaking of gender.

[26] Souza J (2018). A classe média no espelho: sua história, seus sonhos e
ilusões, sua realidade.

[27] Siegrist J (2026). Effort-reward imbalance at work and health: review and critical
appraisal of three decades of research. Scand J Work Environ Health..

[28] Deleuze G, Deleuze G (1992). Post-scriptum sobre as sociedades de controle. Conversações.

[29] Jahnke SA, Haddock CK, Jitnarin N, Kaipust CM, Hollerbach BS, Poston WSC (2019). The prevalence and health impacts of frequent work discrimination
and harassment among women firefighters in the US fire
service. Biomed Res Int..

[30] Stanley IH, Hom MA, Chu C, Dougherty SP, Gallyer AJ, Spencer-Thomas S (2019). Perceptions of belongingness and social support attenuate PTSD
symptom severity among firefighters: a multistudy
investigation. Psychol Serv..

